# Clinical significance of *NF2* alteration in grade I meningiomas revisited; prognostic impact integrated with extent of resection, tumour location, and Ki-67 index

**DOI:** 10.1186/s40478-022-01377-w

**Published:** 2022-05-15

**Authors:** Yu Teranishi, Atsushi Okano, Satoru Miyawaki, Kenta Ohara, Daiichiro Ishigami, Hiroki Hongo, Shogo Dofuku, Hirokazu Takami, Jun Mitsui, Masako Ikemura, Daisuke Komura, Hiroto Katoh, Tetsuo Ushiku, Shumpei Ishikawa, Masahiro Shin, Hirofumi Nakatomi, Nobuhito Saito

**Affiliations:** 1 Department of Neurosurgery, Faculty of Medicine, The University of Tokyo, 7-3-1 Hongo, Bunkyo-ku, Tokyo, 113-8655 Japan; 2 Department of Molecular Neurology, Graduate School of Medicine, The University of Tokyo, 7-3-1 Hongo Bunkyo-ku, Tokyo, Japan; 3 Department of Pathology, Faculty of Medicine, The University of Tokyo, 7-3-1 Hongo, Bunkyo-ku, Tokyo, Japan; 4 Department of Preventive Medicine, Graduate School of Medicine, The University of Tokyo, Tokyo, Japan; 5 Department of Neurosurgery, Faculty of Medicine, Teikyo University, 2-11-1 Kaga, Itabashi-ku, Tokyo, Japan; 6 Department of Neurosurgery, Faculty of Medicine, Kyorin University, 6-20-2 Shinkawa, Mitaka-shi, Tokyo, Japan

**Keywords:** Meningioma genomics, WHO grade, Tumor location, Tumor prognosis, Precision medicine

## Abstract

*NF2* alteration is the most commonly–found genetic abnormality in meningiomas and is known to initiate events for aggressive-type meningiomas. Whereas the prognosis of meningiomas differs depending on their epigenomic/transcriptomic profile, the effect of *NF2* alteration on the prognosis of benign meningiomas is not fully elucidated. This study aimed to probe the importance of *NF2* alteration in prognosis of WHO grade I meningiomas. A long-term retrospective follow-up (5.3 ± 4.5 years) study involving 281 consecutive WHO grade I meningioma patients was performed. We assessed tumour recurrence in correlation with extent of resection (EOR), histopathological findings, tumour location, and *NF2* alteration. “NF2 meningioma” was defined as meningiomas with presence of *NF2* mutation and/or 22q loss. Overall, NF2 meningioma per se was not a predictor of prognosis in the whole cohort; however, it was a predictor of recurrence in supratentorial meningiomas, together with EOR and Ki-67. In a striking contrast, NF2 meningioma showed a better prognosis than non-NF2 meningioma in infratentorial lesion. Supratentorial NF2 meningiomas had higher Ki-67 and forkhead box protein M1 expression than those of others, possibly explaining the worse prognosis in this subtype. The combination of *NF2* alteration, high Ki-67 and supratentorial location defines subgroup with the worst prognosis among WHO grade I meningiomas. Clinical connotation of *NF2* alteration in terms of prognosis of WHO grade I meningioma differs in an opposite way between supratentorial and infratentorial tumors. Integrated anatomical, histopathological, and genomic classifications will provide the best follow-up schedule and proactive measures.

**Supplementary Information:**

The online version contains supplementary material available at 10.1186/s40478-022-01377-w.

## Introduction

Meningiomas are the most common primary central nervous system tumours in the adult population, accounting for approximately 30% of intracranial tumours [1, 2]. Meningiomas originate at the meningeal arachnoid leaf, and are histologically benign in the majority of cases (75% are grade I per WHO classification) [3]. The remaining 25% of meningiomas, categorized as grade II or III per WHO classification, show a rapid growth and high recurrence rate (in WHO grade II, roughly 50% recurrence at 10 years, in WHO grade III, almost 100% recurrence at 10 years) [3]. In general, a 2nd surgery or radiotherapy are common complementary treatment practices for recurrent meningiomas in addition to surgical resection, especially in case of WHO grade II or III meningiomas [3]. However, WHO grade I meningiomas are also known to recur, with a sobering report of around 24–60% recurrence rate with long-term follow-up [1, 3–8]. Despite these reports, observation with or without imaging follow-ups remains commonplace following gross total resection (GTR) and subtotal resection (STR) in WHO grade I meningioma [1]. Thus, for the therapeutic management of WHO grade I meningiomas, identification of cases that are at a higher risk of and of the indicators of recurrence is crucial for making a right decision at the right timing in performing the complementary treatment.

According to previous reports, the extent of resection (EOR), Ki-67 index, and WHO grades based on histological findings are known as predictors of meningioma recurrence [4–13]. Many of these studies have focused on the clinical characteristics of atypical or malignant meningiomas, while recurrence of WHO grade I meningiomas has not yet been fully elucidated [13, 14].

Recent molecular analyses have shown that meningiomas are not genomically homogenous but have various genetic, epigenetic, and transcriptomic profiles [16–25]. For example, driver gene mutations are known to be highly dependent on the anatomical location of meningiomas [16–21]. *Neurofibromatosis 2* (*NF2*) alteration is the most common genetic driver in sporadic meningiomas and is known to initiate events for aggressive-type meningiomas. However, the effect of driver gene mutations, especially *NF2* alteration, on the prognosis of meningiomas has not been clearly established, except a few reports stating that their prognosis in all WHO grade meningiomas differs depending on the epigenomic/transcriptomic profiles [26–31]. Furthermore, tumour location has to be taken into account in considering recurrence of WHO grade I meningiomas because the location of the tumour is also related to its epigenetic profile [24, 32].

In this study, we aimed to investigate the clinical significance of *NF2* alteration in the prognosis of WHO grade I meningiomas. We performed a long-term follow-up study to validate the effect of EOR, the significance of *NF2* alteration, and tumour anatomical location on meningioma recurrence.

## Materials and methods

### Patient population

Patients who underwent surgical resection of sporadic meningioma between 2000 and 2019 were retrospectively queried using an institutional database. The study protocol was approved by our Institutional Review Board (G10028), and informed consent was obtained from all the patients. Patients with incomplete clinical or genetic data, any previous history of meningioma treatment, or any history of radiation therapy for the remaining tumour immediately after the first surgery, were excluded. Patients with WHO grade I meningioma were included in this study (Additional file 1: Figure S1).

### Clinical data

All clinical data were collected through a retrospective chart review. The collected clinical endpoints included patient age, sex, and radiological follow-up. Preoperative and postoperative radiological data including tumour size, anatomical location, EOR, and the presence/absence and timing of recurrence were noted. Patients were followed-up with contrast-enhanced MRI (CE-MRI) within 2 days, around 6 months, and 1 year after surgery. If there was no tumour recurrence, follow-up was continued annually by CE-MRI. We defined tumour recurrence as apparent enlargement of the residual tumour on CE-MRI, by blind inter-observer agreement between the neuro-radiologists and neurosurgeons in charge.

The EOR was categorized as either GTR, which included Simpson grades I, II, and III, or STR, which included Simpson grades IV. The EOR was determined based on the postoperative imaging and operation records.

### Histopathological data

The pathological diagnosis was established by the expert neuropathologist at our institution based on the 2016 WHO classification of tumours of the central nervous system. Formalin-fixed paraffin-embedded tissue was used for immunohistochemistry (IHC) in all cases. IHC was performed using whole-slide sections for forkhead box protein M1 (FOXM1) and Ki-67. The following antibodies were used: anti-Ki67 rabbit polyclonal (30–9; Ventana Medical Systems, Tucson, AZ) and FOXM1 rabbit monoclonal (EPR17379). Ki-67 index was calculated as the highest value in areas of maximum cellular density, identified by visual inspection. We define 4% as the cutoff of Ki-67 in reference to previous publications about Ki-67 and the recurrence of meningioma [33, 34]. FOXM1 IHC was quantified as the highest number of positive nuclei / high power field (HPF) within each meningioma using ImageJ (U.S. National Institutes of Health). “High” FOXM1 expression was defined as more than 3 nuclei/HPF, and “low” as otherwise.

### Sanger sequencing and microsatellite analysis

Tumour samples were stored at −80 °C after tumour resection until genomic analysis. Tumour DNA was obtained from frozen samples using a DNA Extraction Mini Kit (Qiagen, Hilden, Germany) according to the manufacturer’s protocol. Mutation analysis was performed as previously described, including direct Sanger sequencing and microsatellite analysis [35].

### Anatomical groups

Tumour location was defined as the location of the dural attachment of the tumour based on intraoperative observation as well as preoperative imaging. All meningiomas were classified into two groups, namely supratentorial or infratentorial lesions. Supratentorial lesions included meningiomas at the convexity, falx, parasagittal, sphenoid ridge, anterior fossa, middle fossa, clinoid, tuberculum sellae, and cavernous sinus. Infratentorial lesions included meningiomas at the clivus, petro-clivus, petrous, cerebellopontine angle, cerebellar convexity, jugular foramen, and foramen magnum. Tentorial meningiomas were divided into two groups depending on the dominant tumour protrusion direction, as evaluated using sagittal MRI images.

### Subgroups encompassing driver gene mutation and tumour location

“NF2 meningioma” was defined as meningiomas with the presence of *NF2* mutation and/or 22q loss [14]. Based on the driver gene mutation profile and the tumour location, meningiomas of all enrolled patients were categorized into the following four subgroups: “Supratentorial NF2”, “Infratentorial NF2”, “Supratentorial non–NF2”, and “Infratentorial non–NF2”. For each group, we evaluated clinical, radiological, and histopathological data, and progression-free survival (PFS).

### Statistical analysis

Statistical analyses were performed using R version 3.1.2 (R Core Team, http://www.R-project.org). Numerical variables were expressed as the mean and standard deviation. Categorical data were compared between the subgroups using Fisher’s exact test. PFS was evaluated using the Kaplan–Meier method, followed by the log-rank test for each variable.

The Mann–Whitney U test was used to compare two non-parametric continuous variables.

The hazard ratio (HR) of all variables for the presence of recurrence as endpoints was analyzed using the univariate Cox proportional hazards model. Variables associated with endpoints in univariate analyses (p < 0.05) were included in a backward, stepwise manner using p-value multivariate analysis.

All reported p-values were two-sided, and in all comparisons, p-values of less than 0.05, were considered significant. Post-hoc pairwise comparisons between subgroups and prognostic grades were adjusted using the Bonferroni method.

### Data availability

The authors confirmed that the data supporting the findings of this study will be shared upon request from any qualified investigator.

## Results

### Clinical characteristics

A total of 343 patients who underwent surgical treatment for sporadic meningioma were enrolled in this study at the University of Tokyo Hospital between 2000 and 2019 (Additional file 1: Fig. S1). The remaining 281 patients with WHO grade 1 meningioma were eligible for subsequent analyses. The average follow-up period after surgery was 5.3 ± 4.5 years (Table 1). Supratentorial and infratentorial meningiomas accounted for 188 (66.9%) and 93 (33.1%) patients, respectively (Table 1, Table 2). The detailed tumour locations are shown in Additional file 1: Table S1.

**Table 1:** Patient characteristics

Variable	N = 281	Non–recurrent (N = 236)	Recurrent (N = 45)
*Age*	57.4 ± 13.2	58.4 ± 12.4	52.5 ± 16.2
*Sex*	Female: 209 (74.4%)	Female: 181 (76.7%)	Female: 28 (62.2%)
*Follow–up (years)*	5.3 ± 4.5	5.6 ± 4.6	4.1 ± 3.7
*Tumor location*			
Supratentorial lesion	188 (66.9%)	152 (64.4%)	36 (80.0%)
Infratentorial lesion	93 (33.1%)	84 (35.6%)	9 (20.0%)
*Extent of resection*			
GTR	236 (84.0%)	209 (88.6%)	27 (60.0%)
STR	45 (16.0%)	27 (11.4%)	18 (40.0%)
*Ki-67 index*	2.5 ± 2.0	2.2 ± 1.6	3.7 ± 3.2
*Driver gene mutation*			
“NF2” (*NF2* variant or 22q loss)	152 (54.1%)	121 (51.3%)	31 (68.9%)
“Non-NF2”	129 (45.9%)	115 (48.7%)	14 (31.1%)
*Subgroups encompassing driver gene mutation and tumor location*			
“Supratentorial NF2”	109 (38.8%)	79 (33.5%)	30 (66.7%)
“Infratentorial NF2”	43 (15.3%)	42 (17.8%)	1 (2.2%)
“Supratentorial non–NF2”	79 (28.1%)	73 (30.9%)	6 (13.3%)
“Infratentorial non-NF2”	50 (17.8%)	42 (17.8%)	8 (17.8%)

**Table 2:** Comparing variables depending on driver gene mutation, tumor location, and subgroups

Variables		Non-recur	Recur	*p*	*NF2*	Non–*NF2*	*p*	Supratent	Infratent	*p*	Sup. NF2	Inf. NF2	Sup. nNF2	Inf. nNF2	***p***
*In all tumor*	N = 281	N = 236	N = 45		N = 150	N = 131		N = 188	N = 93		N = 109	N = 44	N = 79	N = 49	
5-yr PFS	83.4%				77.9%	90.3%	0.04	82.1%	86.7%	0.18	72.8%	94.1%	95.3%	80.6%	6.2 × 10^–4^
GTR	236 (84.0%)	209 (88.6%)	27 (60.0%)	1.5 × 10^–5^	132 (88.0%)	104 (79.3%)	0.19	158 (84.0%)	78 (83.9%)	1.0	91 (83.5%)	41 (93.2%)	67 (84.8%)	37 (75.5%)	0.04
NF2	152 (54.1%)	121 (51.3%)	31 (68.9%)	0.03				109 (58.0%)	43 (46.2%)	0.07					
Supratent	188 (66.9%)	152 (64.4%)	36 (80.0%)	0.05	109 (72.7%)	79 (60.3%)	0.07								
Ki-67 index	2.5 ± 2.0	2.2 ± 1.6	3.7 ± 3.2	0.01	2.9 ± 2.4	2.0 ± 1.4	0.004	2.7 ± 2.3	2.0 ± 1.3	0.02	3.2 ± 2.6	2.2 ± 1.4	2.0 ± 1.5	1.8 ± 1.2	8.4 × 10^–5^
*In GTR*	N = 236	N = 209	N = 27		N = 131	N = 105		N = 158	N = 78		N = 90	N = 42	N = 68	N = 36	
5-yr PFS	86.9%				81.5%	94.1%	0.02	84.4%	93.1%	0.01	77.3%	93.8%	94.4%	92.3%	6.2 × 10^–3^
NF2	131 (55.5%)	110 (52.6%)	21 (77.8%)	0.01				90 (57.0%)	41 (52.6%)	0.57					
Supratent	158 (66.9%)	133 (63.6%)	25 (92.6%)	0.001	90 (68.7%)	68 (64.8%)	0.57								
Ki-67 index	2.5 ± 2.0	2.3 ± 1.6	4.3 ± 3.4	0.002	2.8 ± 2.3	2.1 ± 1.4	0.008	2.7 ± 2.3	2.0 ± 1.2	0.03	3.2 ± 2.6	2.1 ± 1.3	2.2 ± 1.6	1.8 ± 1.2	0.01
*In STR*	N = 45	N = 27	N = 18		N = 20	N = 25		N = 30	N = 15		N = 18	N = 2	N = 12	N = 13	
5-yr PFS	65.4%				55.6%	75.1%	0.21	69.3%	58.2%	0.61	50.0%	100%	100%	51.3%	0.05
NF2	20 (44.4%)	10 (37.0%)	10 (55.6%)	0.2				18 (60.0%)	2 (13.3%)	0.004					
Supratent	30 (66.7%)	19 (70.4%)	11 (61.1%)	0.53	18 (90.0%)	12 (48.0%)	0.004								
Ki-67 index	2.3 ± 2.1	1.9 ± 1.7	2.8 ± 2.7	0.42	3.3 ± 2.6	1.5 ± 1.2	0.01	2.4 ± 2.3	2.0 ± 1.7	0.6	3.3 ± 2.6	3.5 ± 3.5	1.2 ± 0.9	1.7 ± 1.4	0.1

Recur.: Recurrence, NF2 meningioma: meningioma with presence of *NF2* variant or 22q loss, Supratent.: Supratentorial, Infratent.: Infratentorial, Sup. NF2: Supratentorial NF2, Inf. NF2: Infratentorial NF2, Sup. nNF2: Supratentorial non–NF2, Inf. nNF2: Infratentorial non–NF2, 5-yr PFS: 5-year progression free survival, GTR: gross total resection, STR:subtotal resection

### Surgical outcome

The overall recurrence rate of WHO grade I meningiomas was 16.0% in the follow-up period (Table 1 & Table 2), and the average time for recurrence was 4.1 ± 3.7 years (Additional file 1: Table S2). 5-year PFS was 83.4% (CI 77.3–88.1), and GTR was achieved in 84.0% of patients (Tables 1, 2).

### Driver gene mutation and subgroups

Molecular analysis revealed that there were 152 NF2 meningiomas (54.1%) and non-NF2 meningiomas: 45.9% (129 cases) including “*AKT1*”: 11.4% (32 cases), “*KLF4*”:5.7% (16 cases), “*POLR2A*”:16 cases (5.7%), “*SMO*”: 0.7% (2 cases), and others:22.1% (62 cases) (Table 1, Additional file 1: Table S1). Based on the mutation and anatomical location, the cases were classified into the following subgroups: “Supratentorial NF2”, 109 cases (38.8%); “Infratentorial NF2”, 43 cases (15.3%); “Supratentorial non–NF2”, 79 cases (28.1%); and “Infratentorial non–NF2”, 50 cases (17.8%) (Table 1).

### Association of the prognosis with EOR, tumour location, driver gene mutation, and subgroups.

**Fig. 1:**
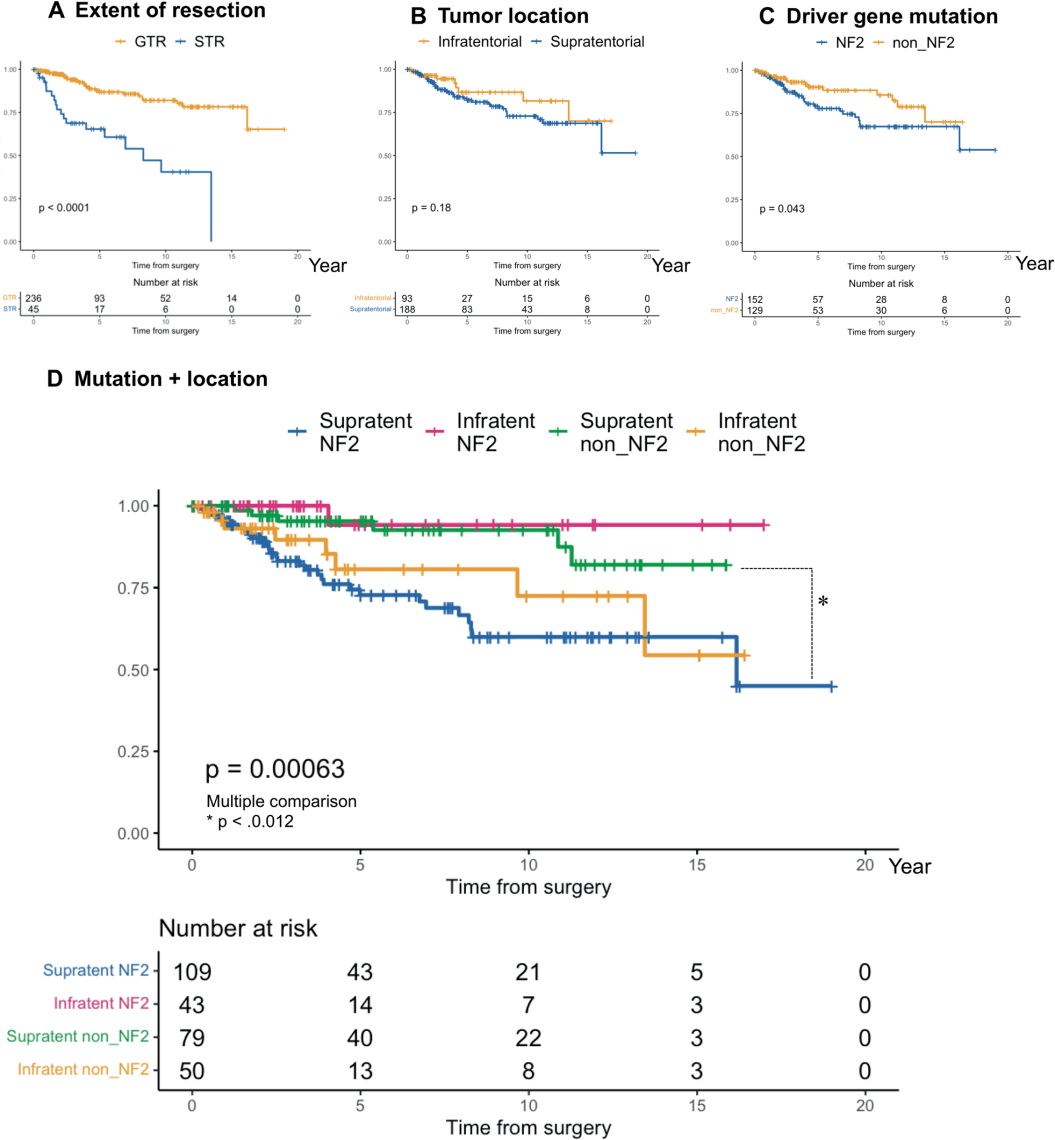
PFS of patients with WHO grade I meningioma evaluated using the Kaplan–Meier method followed by the log-rank test for each variable, **A**: EOR, **B**: tumour location, **C**: driver gene mutation, **D**: subgroups with mutation and location. EOR: the extent of resection; PFS: progression-free survival

Consistent with previous reports, the cases with GTR had a better prognosis than those with STR (5-year PFS: 86.9% in GTR vs. 65.4% in STR, p = 8.4 × 10^–8^) (Table 2, Fig. 1A) [4–13]. We found that the prognosis of the meningiomas was not different location-wise (5-year PFS: 82.1% in “Supratentorial” vs 86.7% in “Infratentorial”, p = 0.18) (Table 2, Fig. 1B) but was different mutation-wise (5-year PFS: 77.9% in *NF2* vs. 90.3% in non-*NF2*, p = 0.04 (Table 2, Fig. 1C). By subgrouping with mutation and location, they became more distinctively different (5-year PFS: 72.8% in “Supratentorial NF2” vs. 94.1% in “Infratentorial NF2” vs 95.3% in “Supratentorial non–NF2” vs 80.2% in “Infratentorial non–NF2”, p = 6.2 × 10^–4^) (Table 2, Fig. 1D). In supratentorial lesion, the prognosis of NF2 meningioma was worse than that of non-NF2 meningioma. In infratentorial lesion, however, the prognosis of NF2 meningioma was better than that of non-NF2 meningioma (Table 2, Fig. 1D).

#### Meningiomas with GTR or STR

**Fig. 2:**
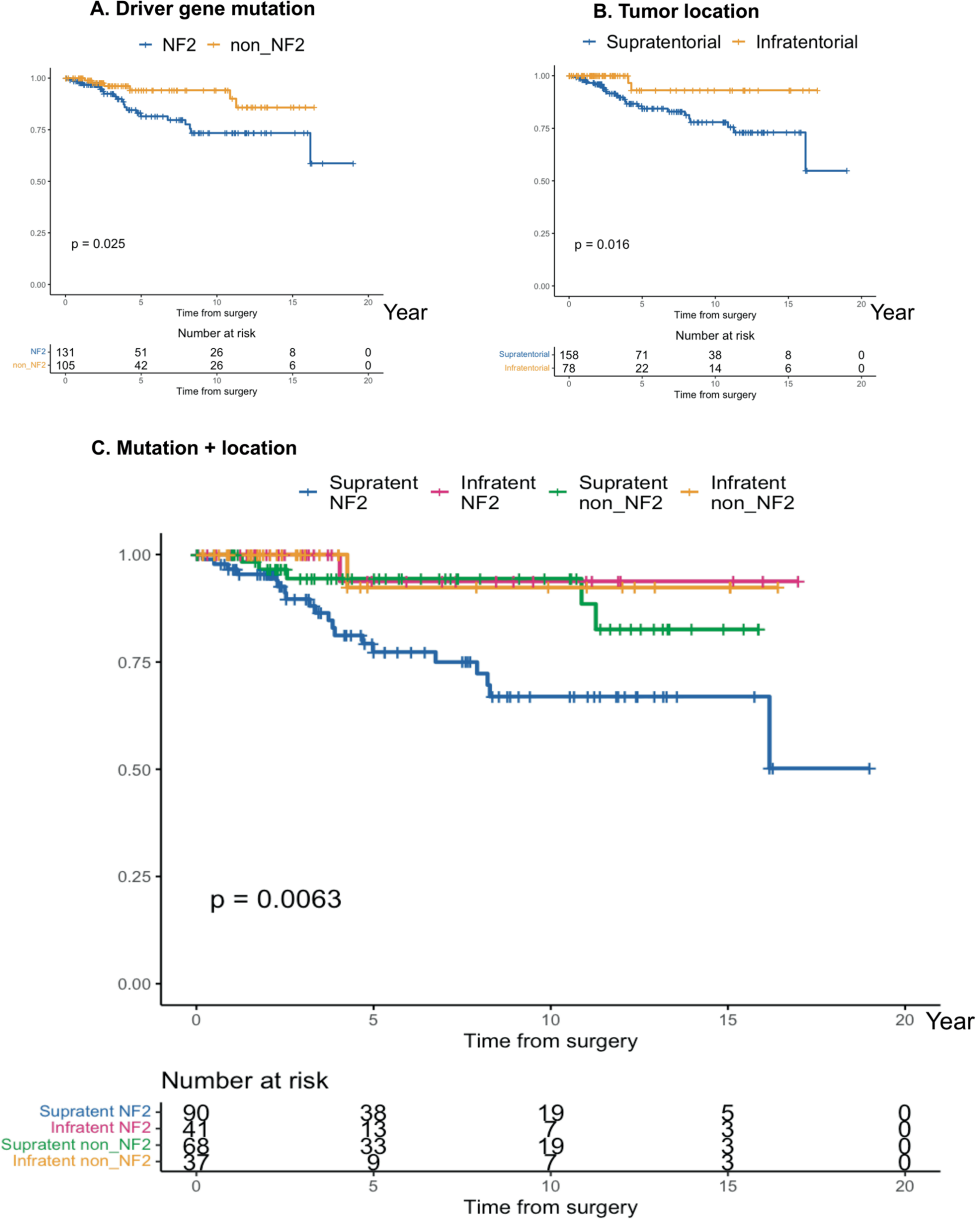
PFS of patients with WHO grade I meningioma with GTR evaluated using the Kaplan–Meier method followed by the log-rank test for each variable, **A**: driver gene mutation, **B**: tumour location, **C**: subgroups with mutation and location. GTR: gross total resection; PFS: progression-free survival

To avoid the effect of EOR on the recurrence, we further compared the prognosis in the meningioma with the same EOR (GTR or STR). Among tumors with GTR, the prognosis was better in non-NF2 meningioma (5-year PFS; 81.5% in NF2 meningioma vs. 94.1% in non-NF2 meningioma; p = 0.02) and also better in infratentorial meningioma (5-year PFS; 84.4% in “Supratentorial” vs. 93.1% in “Infratentorial”; p = 0.01) (Table 2, Fig. 2A, B). By subgrouping, the prognosis in the “Supratentorial NF2” subgroup was worst (5-year PFS: 77.3% in “Supratentorial NF2” vs. 93.8% in “Infratentorial NF2” vs. 94.4% in “Supratentorial non–NF2” vs. 92.3% in “Infratentorial non–NF2”, p = 6.2 × 10^–3^) (Table 2, Fig. 2C).

In contrast, in tumors with STR, the prognosis in “Supratentorial NF2” remained worse, but 5-year PFS in “Infratentorial non–NF2” was also low (5-year PFS: 50.0% in “Supratentorial NF2”, 100% in “Infratentorial NF2”, 100% in “Supratentorial non–NF2”, 51.3% in “Infratentorial non–NF2”; p = 0.05) (Table 2).

### Histopathological findings and their correlation to prognosis

**Fig. 3:**
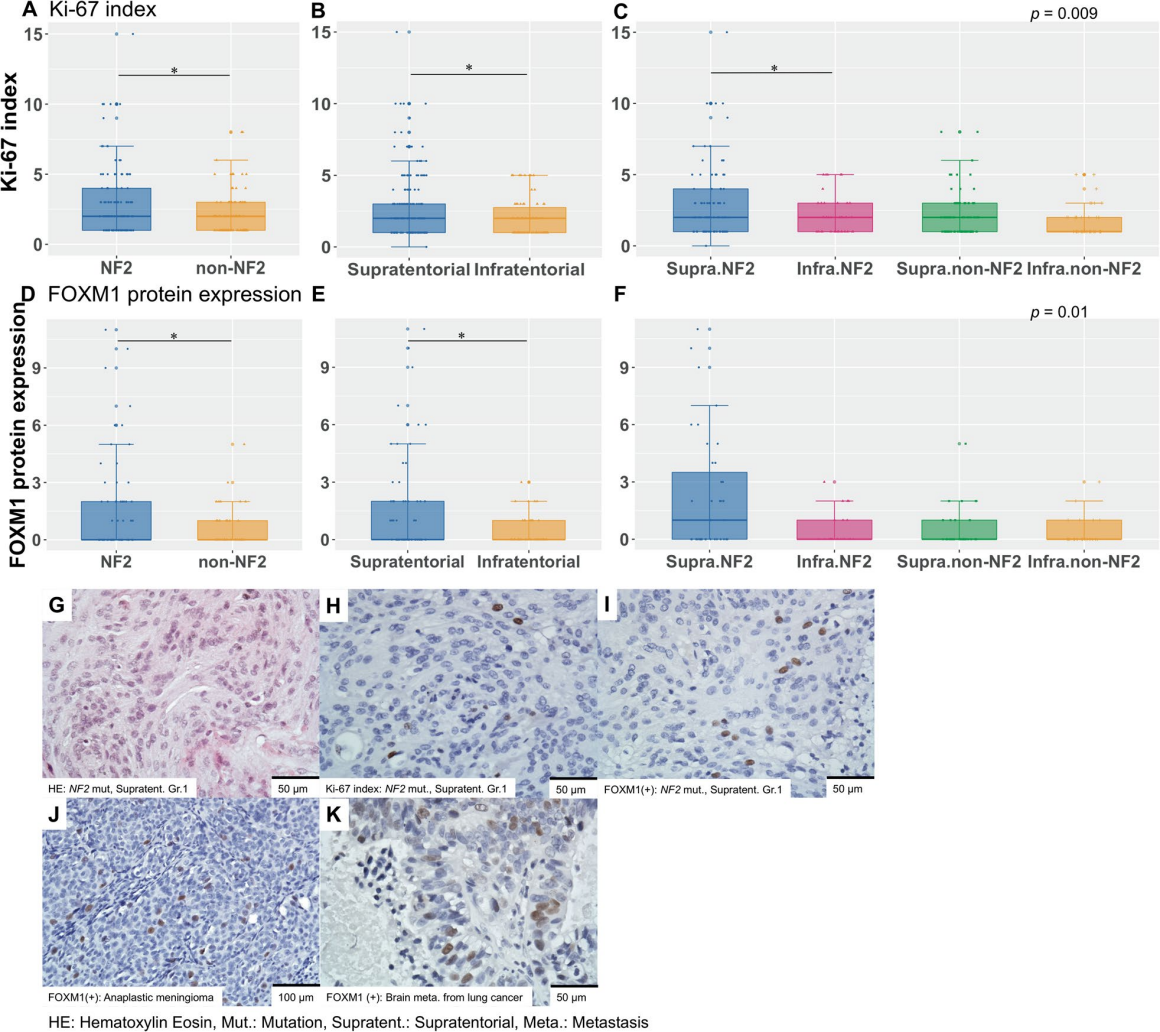
Ki-67 index and FOXM1 protein expression in WHO grade I meningioma with GTR. Comparison of Ki-67 index based on each variable (**A**: driver gene mutation, **B**: tumour location, **C**: subgroups). Comparison of FOXM1 protein expression based on each variable (**D**: driver gene mutation, **E**: tumour location, **F**: subgroups). **G**: hematoxylin eosin (WHO grade I meningioma in supratentorial lesion with *NF2* mutation, scale bar: 50 µm). **H**: Ki-67 index (WHO grade I meningioma in supratentorial lesion with *NF2* mutation, scale bar: 50 µm). **I**: FOXM1 protein expression (WHO grade I meningioma in supratentorial lesion with *NF2* mutation, scale bar: 50 µm). **J**: FOXM1 protein expression (WHO grade III anaplastic meningioma, scale bar: 100 µm). **K**: FOXM1 protein expression (brain metastasis from lung cancer, scale bar: 50 µm). FOXM1: forkhead box protein M1; WHO: World Health Organization. GTR: gross total resection; PFS: progression-free survival

The histopathological findings of meningiomas are shown in Fig. 3 and Additional file 1: Table S1, and they are consistent with previous reports [1]. The prognosis was found to be different depending on Ki-67 index (Ki-67 ≥ 4 vs. Ki-67 < 4, p = 4.5 × 10^–9^). Among meningiomas with GTR, PFS was also different depending on Ki-67 index (Ki-67 ≥ 4 vs. Ki-67 < 4, *p* = 4.9 × 10^–10^), FOXM1 protein expression in 111 cases (high vs. low, *p* = 0.00052), and FOXM1 protein expression in 40 cases of supratentorial NF2 meningioma (high vs. low, *p* = 0.013) (Additional file 1 Fig. S2A, 2B, 2C).

### HR for PFS in WHO grade I meningiomas

**Fig. 4:**
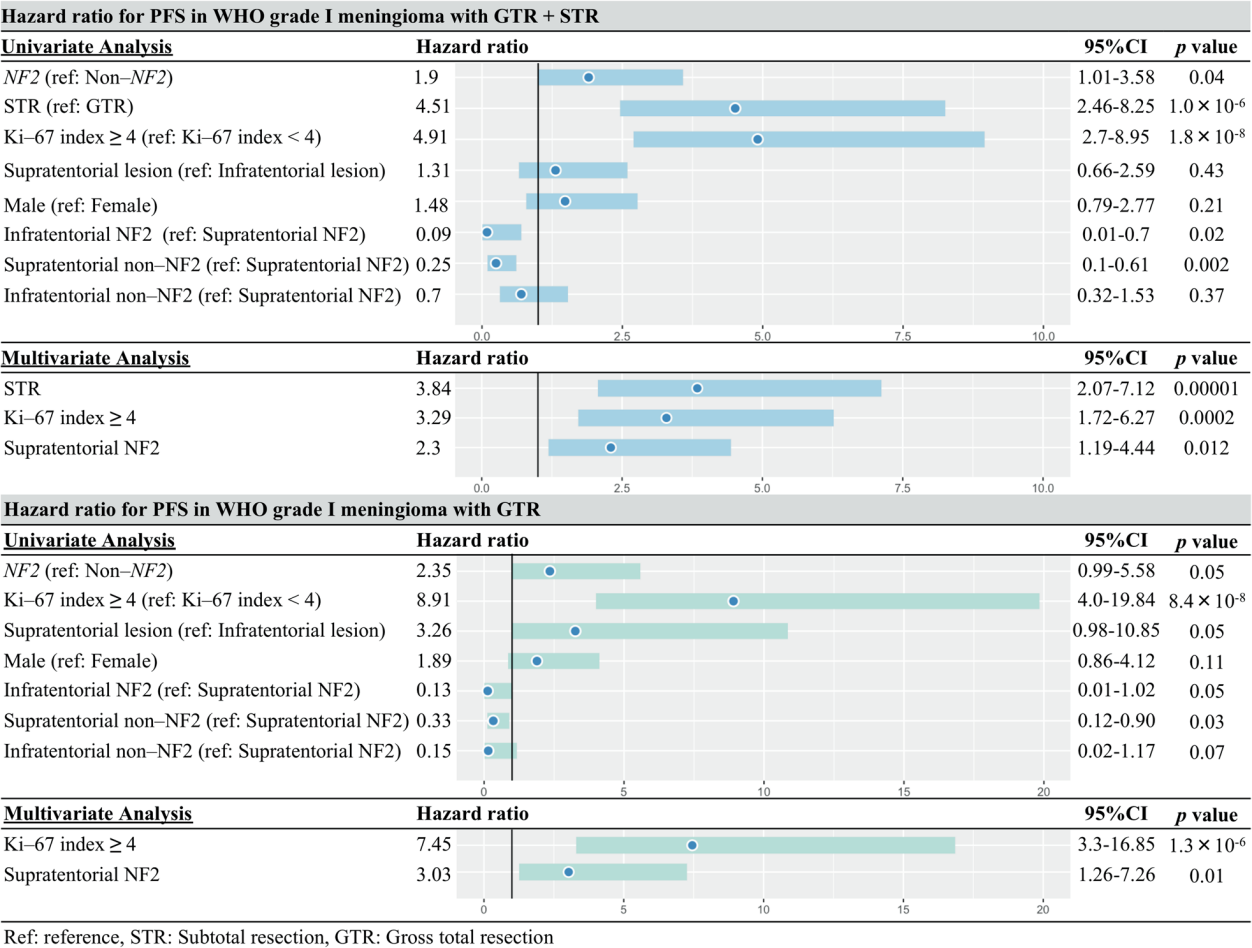
Hazard ratio for PFS in WHO grade I meningiomas (upper: in all tumors, lower: in tumor with GTR)

In univariate analyses, statistical associations were identified between worse prognosis and NF2 meningioma (HR:1.9, confidence interval [CI] 1.01–3.58, p = 0.04), STR (HR: 4.51, CI 2.46–8.25, p = 1.0 × 10^–6^), and Ki-67 index ≥ 4 (HR: 4.91, CI 2.7–8.95, p = 1.8 × 10^–8^) (Fig. 4). In subgroups (reference to “Supratentorial NF2), “Infratentorial NF2” (HR: 0.09, CI: 0.01–0.7, p = 0.02), “Supratentorial non–NF2” (HR: 0.25, CI 0.1–0.61, p = 0.002) showed longer PFS.

Multivariate analysis showed that NF2 meningioma per se was not proven to be a predictor of prognosis. However, STR (HR: 3.84, CI 2.07–7.12, p = 0.00001), Ki-67 index ≥ 4 (HR: 3.29, CI 1.76–6.27, p = 0.0002), and “Supratentorial NF2” (HR: 2.3, CI 1.19–4.44 p = 0.012) were found to be independent significant predictors of recurrence on multivariate analysis (Fig. 4).

In univariate analyses for the meningiomas with GTR, statistical associations were found between worse prognosis and identified with Ki-67 index ≥ 4 (HR: 8.91, CI 4.0–19.84, p = 8.4 × 10^–8^) (Fig. 4). In subgroups (reference to “Supratentorial NF2), “Supratentorial non–NF2” (HR: 0.33, CI: 0.12–0.90, p = 0.03) were positively associated with PFS.

In WHO grade I meningiomas with GTR, tumour location (supratentorial or infratentorial), *NF2* status (presence or absence) and Ki-67 index (≥ 4 or not) were used as covariates in the multivariate analysis (Fig. 4). FOXM1 expression was omitted, as it is involved in the cell cycle and is assumingly closely associated with Ki-67 index [31]. Ki-67 index ≥ 4 (HR: 7.45, CI: 3.3–16.85, p = 1.3 × 10^–6^), and “Supratentorial NF2” (HR: 3.03, CI: 1.26–7.26 p = 0.01) were found to be significant predictors of prognosis.

### Classifications of WHO grade I meningioma with GTR based on Ki-67 index, driver gene mutation, and tumour location.

**Fig. 5:**
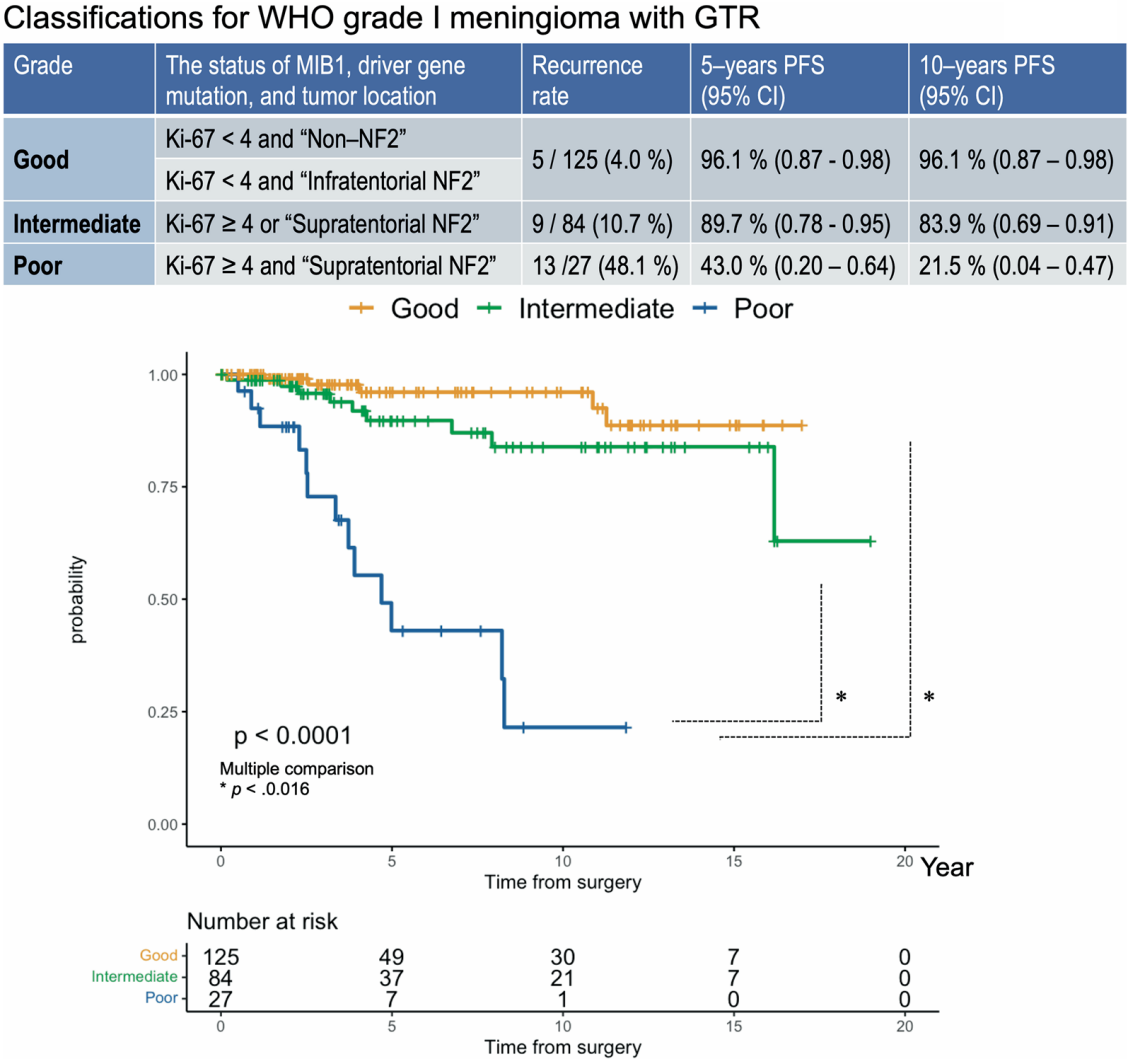
Classifications of WHO grade I meningioma with GTR. “Good”: Ki-67 index < 4 and non-NF2 meningioma, Ki-67 index < 4 and “Infratentorial NF2”; Intermediate: MIB-1 index ≥ 4 or “Supratentorial NF2”; Poor: Ki-67 index ≥ 4 and “Supratentorial NF2”

According to the results of the multivariate analysis, we settled classes of WHO grade I meningioma that achieved GTR using three clinical/histopathological/genomic factors as follows: “Good”: Ki-67 index < 4 and non-NF2 meningioma, Ki-67 index < 4 and “Infratentorial NF2”; Intermediate: MIB-1 index ≥ 4 or “Supratentorial NF2”; Poor: Ki-67 index ≥ 4 and “Supratentorial NF2” (Fig. 5). The results in terms of 5-year and 10-year PFS were, Good, 96.1%/ 96.1%; Intermediate, 89.7% /83.9%; and Poor, 43.0%/21.5%. PFS was also different depending on each class (p = 3.8 × 10^–13^) (Fig. 5).

## Discussion

Recent genetic and clinical studies have evaluated clinical/molecular characteristics of meningiomas including all WHO grades [22–31]; however, recurrence of WHO grade I meningiomas, especially after GTR has not been well-studied based on the assumption that benign meningioma with perfect resection should be of paramount reassurance [3, 11]. In the present study, we performed a long-term follow-up study (5.3 ± 4.5 years) for 281 WHO grade I meningiomas. The study revealed the significance of *NF2* mutation and/or 22q loss in WHO grade I meningiomas in predicting recurrence, which was intriguingly reinforced by tumour anatomical locations. By integrating driver gene mutation, tumour location, and Ki-67 index, we now present a novel prognostic three-class category to predict tumour recurrence for WHO grade I meningiomas following GTR.

### Association between driver gene mutation and recurrence in WHO grade I meningiomas

According to the recent study about the association between subgrouping by driver gene mutation and recurrence of meningiomas [14], only the Hedgehog group remained a significant genetic predictor of tumour recurrence by multivariate analysis in all WHO grade meningiomas. Furthermore, regarding WHO grade I meningiomas, the PFS of the group with the Hedgehog and the tumour necrosis factor-receptor associated factor 7 (TRAF 7) were shorter than that of the *NF2* group [14]. In our results, the prognosis of the meningiomas was different mutation-wise (5-year PFS: 77.9% in *NF2* vs 90.3% in non-*NF2*, p = 0.04 (Table 2, Fig. 1C) and univariate analysis identified between worse prognosis and NF2 meningioma (HR:1.9, confidence interval [CI] 1.01–3.58, p = 0.04)”, however, the statistical significance was marginal. Corroborating their results, NF2 meningioma per se was not a significant predictor by multivariate analysis for PFS in WHO grade I meningiomas in the current study. This finding is counterintuitive, considering that *NF2* alteration are known to initiate events for aggressive-type meningiomas [36]. Actually, the latest trends of the molecular subgroups for meningioma focus on whether NF2 meningioma or not according to the data of single-cell RNA sequencing in meningiomas [25, 37, 38].

Addressing this pathophysiological question, the present study probed the connotation of driver gene mutation status in association with tumour location and revealed that the clinical significance of the *NF2* mutation or 22q loss differed remarkably or inversely with tumour location. The presence of *NF2* alteration was found to have a significantly worse prognostic impact in supratentorial meningiomas, while it has a tendency to have a better prognostic effect in infratentorial meningiomas. More specifically, “Supratentorial” and “NF2” was the worst combination as a predictor of PFS in subgroups. And this was echoed by a significantly higher Ki-67 index and FOXM1 expression in this subgroup compared with others.

Recently, meningioma development was reviewed from an embryological perspective by Fountain et al. and Boetto et al. [39, 40]. Collaborating these reports and our result, we speculate that the difference in the prognostic implication of *NF2* alteration between supra- and infratentorial space may be attributed to the spatial distribution of the tumour cell of origin (neural crest vs mesoderm). Correspondingly, an integrated study surrounding location, phenotype, genotype, and meningeal embryology might be meaningful study in the future.

### Association between other variates and recurrence in WHO grade I meningiomas

The EOR has been known as a prognostic factor for meningiomas [4–9], and in clinical practice, the Simpson grading system has been commonly utilized as a landmark to predict recurrence of meningiomas [41]. In our study, the EOR was a strong predictor of tumour recurrence. However, from a neurosurgical viewpoint, the EOR is highly and inevitably dependent on tumour location [24, 32]. Furthermore, the genomic profile is strongly correlated with tumour location [16–21]. Thus, the effect of each variable, including EOR, tumour location, and molecular subgroups, should be equally considered when evaluating meningioma recurrence.

In our results, the GTR/STR rate was not statistically different between supratentorial and infratentorial tumours (Table 2). However, most of the infratentorial tumours in which GTR was not achieved were non-*NF2* meningiomas (4.7% in “Infratentorial NF2” vs. 26.0% in “Infratentorial non–NF2”, p = 0.04) (Table 2). In addition, they were mainly located surrounding brainstem such as at clivus, and petro-clival region (Additional file 1: Fig. S3). Previous studies reported that one of the prognostic factors for meningioma recurrence was posterior fossa [15] and that STR was an independent predictor of recurrence in skull base meningiomas [42]. Our results showed short PFS in infratentorial non–NF2 meningiomas (Fig. 1, Table 2). This result needs careful interpretation; one explanation is that only STR was achieved in many of infratentorial non–NF2 meningiomas due to the surgical difficulty related to their proximity to the cranial nerves, vessels, and brainstem. This contributed to a higher rate of recurrence compared with infratentorial NF2 meningiomas, which are often located at cerebellar convexity.

In contrast, even if GTR was achieved, the 5-year PFS in patient with NF2 meningioma remained short in the supratentorial space (Table 2). We speculated that despite being WHO grade I, the short PFS of these meningiomas reflected the aggressive histological features such as high Ki-67 index and high FOXM1 expression. This result should weigh in our decision-making regarding the appropriate management of WHO grade I supratentorial meningioma after GTR. Actually, the prognosis of “Poor” class WHO grade I supratentorial meningioma was quite similar to that of WHO grade II or III meningiomas (Fig. 5). Facing this sobering reality, we here recommend integrated histopathological and molecular diagnosis, close follow-up, and possibly proactive treatment protocol to be implemented in cases of WHO grade I meningioma with GTR when characterized by *NF2* alteration, supratentorial location, and high Ki-67 index.

Our study had several limitations that should be addressed in future investigations. First, our study suffers from retrospective, single-institution design, which restricted variables for the assessment to those included in the database. Charts were reviewed retrospectively, and thus, not all clinical/genomic data could be collected. The duration of follow-up was not ideally sufficient to evaluate the prognosis of WHO grade I meningiomas, especially considering that the average time to recurrence was only a little shorter than the median follow-up time. As *NF2* mutation was predominantly the commonest mutation in meningioma, we simplified the analyses by categorizing the meningioma as *NF2* or non-*NF2*. In the future, we hope to analyze the effects of several other driver gene mutation types in association with anatomical location in meningioma recurrence. Further external studies on a large number of cases are warranted to validate our results to a better understanding of the clinical behaviour of this disease.

Previously, *NF2* alteration, multiple copy number variations (CNV; e.g. 22q loss, 1p loss, etc.), high FOXM1 expression, low immune cell infiltration, and loss of H3K27me3 were reported as characteristics of aggressive behaviour of meningiomas [28, 43]. Recent reports have speculated structure variants such as multiple CNVs, especially 1p loss, as an epigenetic “second-hit” following *NF2* alteration for aggressive-type meningiomas [22, 24, 38, 45]. Although we posit that supratentorial NF2 meningiomas might have such an aggressive feature as copy number alteration in addition to Ki-67 and FOXM1, it was beyond the scope of this study. Thus, evaluation of CNV status in *NF2* mutated meningiomas depending on anatomical location is also needed in the future.

In terms of evaluating tumour anatomical location, while there are many ways of categorization, here we divided the cases into supra- or infra-tentorium, which possibly reflected the biological differences of the brain tumours, as evidenced by ependymomas [46]. However, more granular anatomical classification should be utilized ideally for clinical utilization. We will focus on the recurrence and the molecular status of meningiomas in each granular anatomical location.

## Conclusion

In conclusion, by conducting this long-term follow-up study of a large number of patients with WHO grade I meningiomas, we demonstrated that the clinical significance of *NF2* alteration status in WHO grade I meningiomas was different depending on tumour anatomical location, i.e., either supratentorial or infratentorial. By integrating driver gene mutation and tumour location, the “Supratentorial NF2” subgroup as well as the EOR and Ki-67 index, were identified as a significant predictor of recurrence of WHO grade I meningioma, clinically similar to the poor prognosis of WHO grade II/III. Correspondingly, even if GTR is achieved in WHO grade I meningiomas, this study suggested that close follow-up and proactive measures should be considered in cases characterized by *NF2* alteration, supratentorial location, and high Ki-67 index. We anticipate that this integrated anatomical, histopathological, and genomic classification carries significant clinical implications and will provide the best follow-up schedule and proactive measures, as well as improve the daily clinical practice for patients with this most common brain tumour.

## Competing interests

The authors report no competing interests.

## Supplementary Information

**Additional file 1.Figure S1**. Flowchart in this study, **Figure S2**. PFS of patients with WHO grade I meningioma with GTR evaluated using the Kaplan-Meier method followed by the log-rank test for each variable, A: Ki-67 index, B: FOXM1 protein expression, C: FOXM1 protein expression in supratentorial NF2 meningiomas. GTR: gross total resection; PFS: progression-free survival; FOXM1: forkhead box protein M1, **Figure S3**. Detailed tumor location WHO grade I meningiomas considering recurrence, and EOR, **Table S1**. Detailed patient characteristics, **Table S2**. Comparing variables depending on driver gene mutation, tumor location, and subgroups
