# Supplementary material for: Clinical significance of NF2 alteration in grade I meningiomas revisited; prognostic impact integrated with extent of resection, tumour location, and Ki-67 index
Source: Acta Neuropathol Commun. 2022 May 15;10:76. doi: 10.1186/s40478-022-01377-w (PMC9107722; doi:10.1186/s40478-022-01377-w)
Supplement: Supplementary file 1 — Additional file 1.Figure S1. Flowchart in this study, Figure S2. PFS of patients with WHO grade I meningioma with GTR evaluated using the Kaplan-Meier method followed by the log-rank test for each variable, A: Ki-67 index, B: FOXM1 protein expression, C: FOXM1 protein expression in supratentorial NF2 meningiomas. GTR: gross total resection; PFS: progression-free survival; FOXM1: forkhead box protein M1, Figure S3. Detailed tumor location WHO grade I meningiomas considering recurrence, and EOR, Table S1. Detailed patient characteristics, Table S2. Comparing variables depending on driver gene mutation, tumor location, and subgroups [file 40478_2022_1377_MOESM1_ESM.pdf]

Figure S1. Flow chart in this study

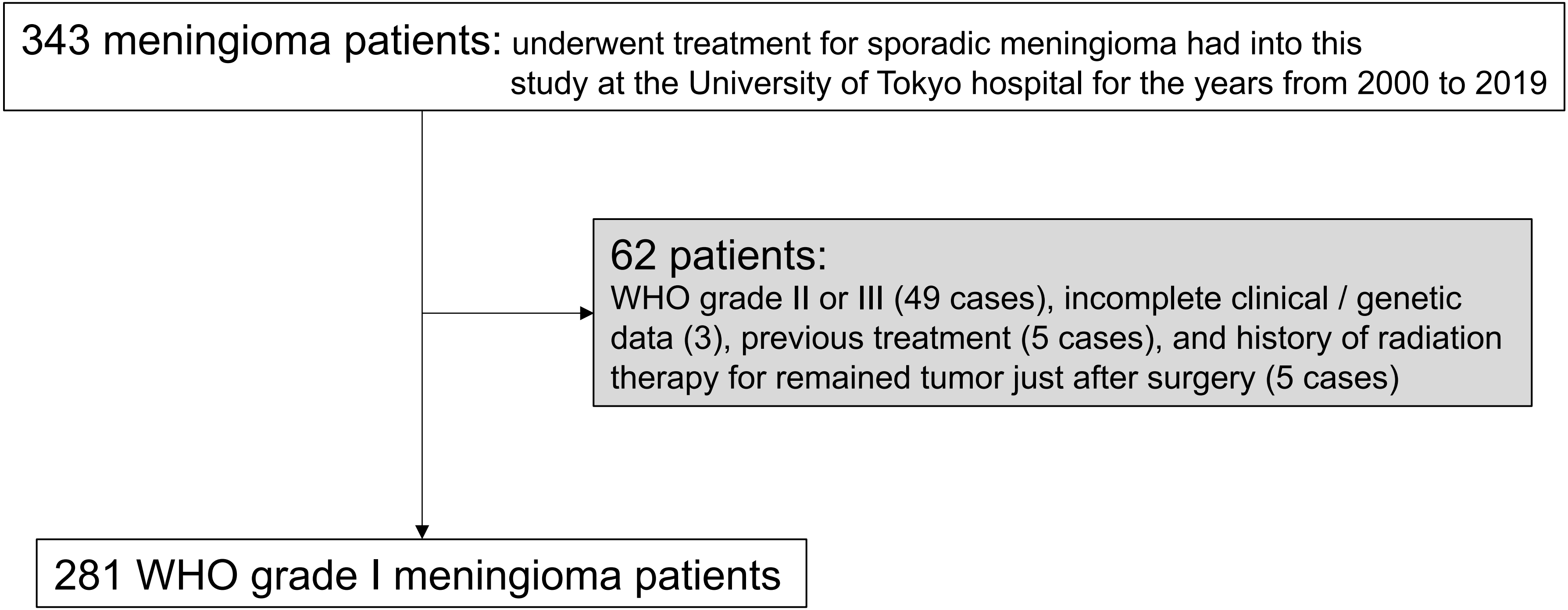

Figure S2

PFS of WHO grade I meningioma with GTR

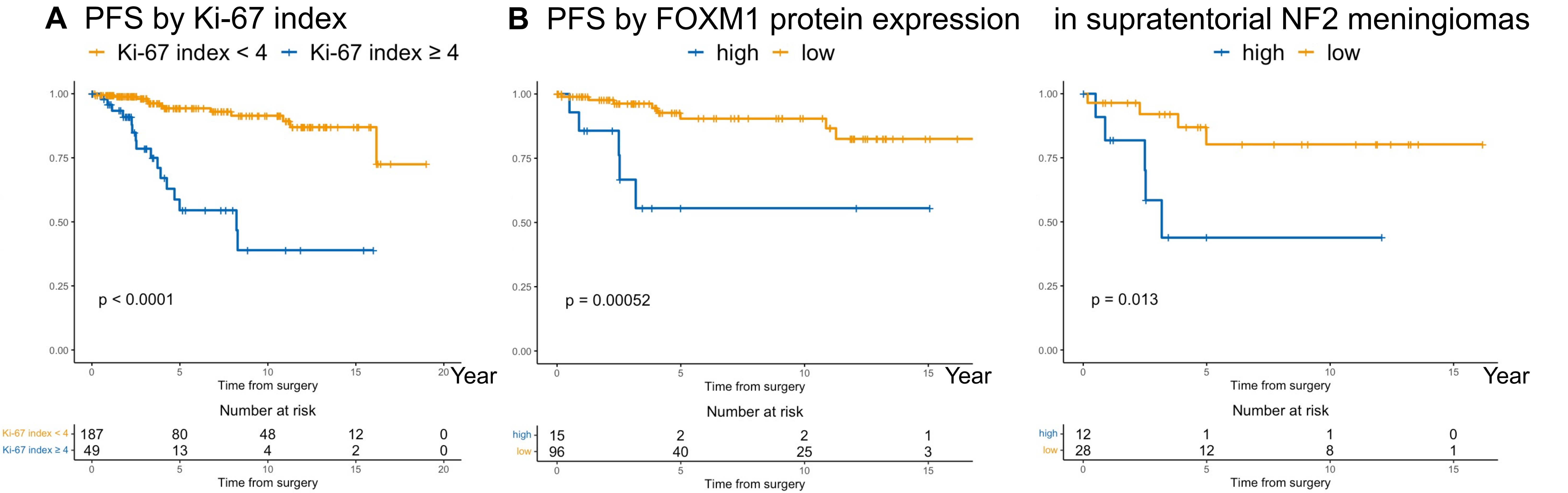

Figure S3
Detailed tumor location WHO grade I meningiomas considering recurrence, and EOR

"NF2" "Non-NF2" : Recurrence : STR

GTR only

GTR and STR

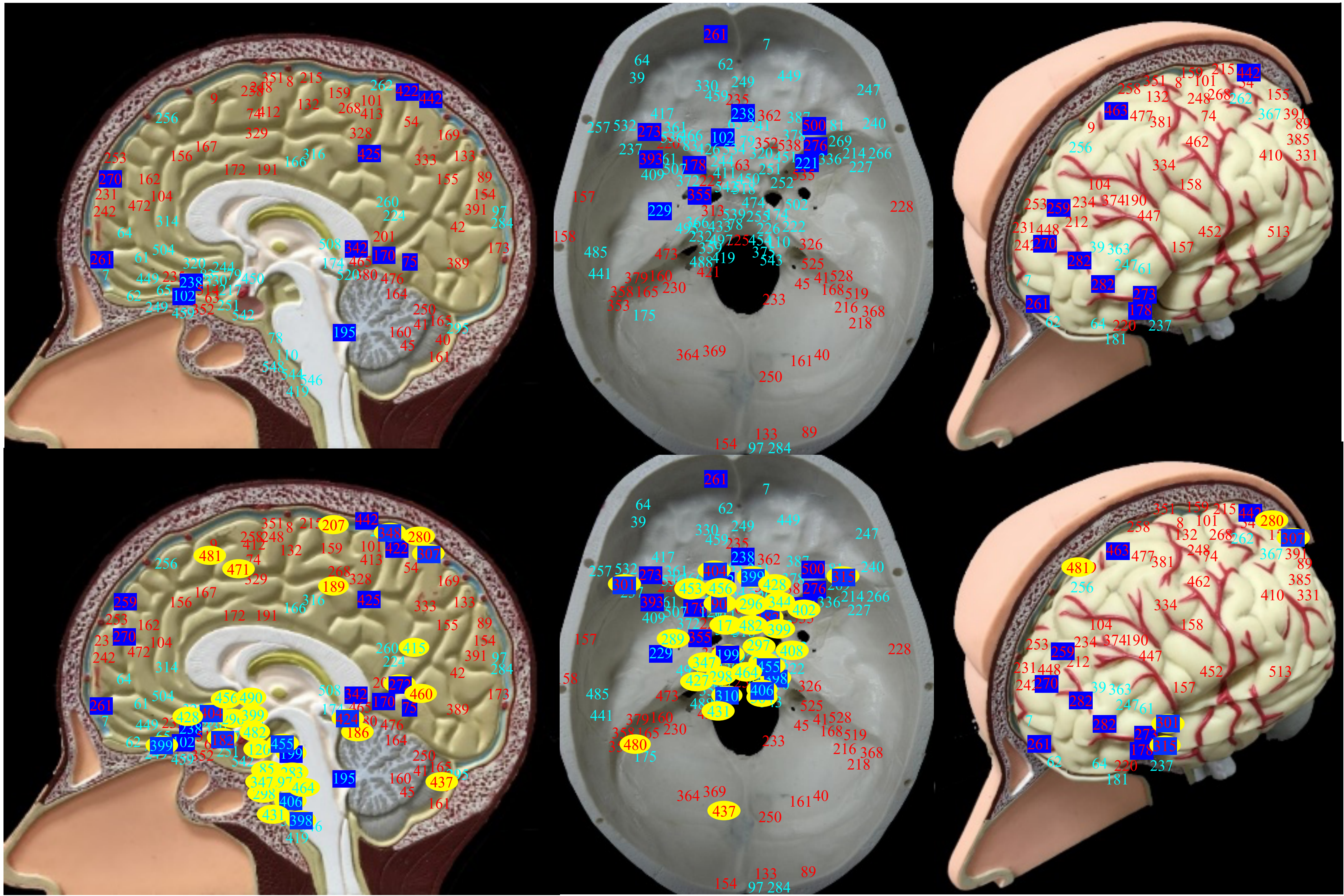

Table S1. Detailed patient characteristics

Variable	N = 281	Non–recurrent (N = 236)	Recurrent (N = 45)
**Supratentorial lesion**	188 (66.9 %)	152 (64.4 %)	36 (80.0 %)
Convexity	37 (13.1 %)	33 (14.0 %)	4 (8.9 %)
Falx, Parasagittal	57 (20.2 %)	49 (20.8 %)	8 (17.8 %)
MSB	50 (17.7 %)	38 (16.1 %)	12(26.7 %)
ASB	29 (10.3 %)	24 (10.2 %)	5 (11.1 %)
Tentorial (Supra)	10 (3.5 %)	5 (2.1 %)	5 (11.1 %)
Ventricular	6 (2.1 %)	5 (2.1 %)	1 (2.2 %)
**Infratentorial lesion**	93 (33.1 %)	84 (35.6 %)	9 (20.0 %)
Cerebellar convexity	9 (3.2 %)	9 (3.8 %)	0 (0 %)
Petroclival, Clivus	22 (7.8 %)	17 (7.2 %)	5 (11.1 %)
Cerebellopontine Angle	34 (12.1 %)	31 (13.1 %)	3 (6.7 %)
Tentorial (Infra)	14 (4.9 %)	14 (5.9 %)	0 (0 %)
Foramen Magnum	8 (2.8 %)	8 (3.4 %)	0 (0 %)
Spinal	3 (1.0 %)	3 (1.3 %)	0 (0 %)
**GTR**	236 (83.9 %)	209 (88.6 %)	27 (60.0 %)
Simpson I	63 (22.4%)	60 (25.4 %)	3 (9.5 %)
Simpson II	140 (49.8%)	124 (52.5 %)	16 (35.6 %)
Simpson III	33 (11.7%)	25 (10.6 %)	8 (17.8 %)
**STR**	45 (16.0%)	27 (11.4 %)	18 (40.0 %)
Simpson IV	45 (16.0%)	27 (11.4 %)	18 (40.0 %)
**Ki-67 index**	2.5 ± 2.0	2.2 ± 1.6	3.7 ± 3.2
Ki-67 index ≥ 4	60 (21.3%)	38 (16.1 %)	22 (48.9 %)
Driver gene mutation			
**“NF2” (*NF2* variant or 22q loss)**	152 (54.1%)	121 (51.3 %)	31 (68.9 %)
**“Non-NF2”**	129 (45.9%)	115 (48.7%)	14 (31.1%)
AKT1	32 (11.4%)	31 (13.1%)	1 (2.2%)
KLF4	16 (5.7%)	14 (5.9%)	2 (4.4%)
POLR2A	16 (5.7%)	12 (5.1%)	4 (8.9%)
SMO	2 (0.7%)	1 (0.4%)	1 (2.2%)
Others	62 (22.1%)	54 (22.9%)	6 (13.3%)

Table S2. Comparing variables depending on driver gene mutation, tumor location, and subgroups

Variables		Non-recur.	Recur.	*p*	*NF2*	Non–*NF2*	*p*	Supratent.	Infratent.	*p*	*NF2*, Sup	*NF2*, Inf	*nNF2*, Sup	*nNF2*, Inf	*p*
In all tumor	N = 281	N = 236	N = 45		N = 150	N = 131		N = 188	N = 93		N = 109	N = 44	N = 79	N = 49	
**5-yr PFS**	83.4%				77.9 %	90.3 %	0.04	82.1 %	86.7 %	0.18	72.8 %	94.1 %	95.3 %	80.6 %	6.2×10^{-4}
**Time to recur. (yr)**	4.1 ± 3.7				3.8 ± 3.3	4.9 ± 4.4	0.55	4.1 ± 3.6	4.4 ± 4.4	0.94	3.8 ± 3.4	4.0	5.5 ± 4.5	4.4 ± 4.7	0.72
**GTR**	236 (84.0 %)	209 (88.6%)	27 (60.0 %)	1.5×10^{-5}	132 (88.0 %)	104 (79.3 %)	0.19	158 (84.0 %)	78 (83.9 %)	1.0	91 (83.5 %)	41 (93.2 %)	67 (84.8 %)	37 (75.5%)	0.04
**Age**	57.4 ± 13.2	58.4 ± 12.4	52.5 ± 16.2	0.02	58.3 ± 12.6	52.9 ± 15.8	0.03	58.4 ± 12.8	55.4 ± 13.9	0.11	59.8 ± 12.8	56.6 ± 13.4	56.6 ± 12.8	54.4 ± 14.3	0.09
**Sex (Female)**	209 (74.3 %)	179 (75.8 %)	28 (66.7 %)	0.19	115 (75.7 %)	94 (72.9 %)	0.68	135 (71.8 %)	74 (79.6 %)	0.19	80 (73.4 %)	35 (81.4 %)	55 (69.6 %)	39 (78.0 %)	0.5
**Follow–up (yr)**	5.3 ± 4.5				5.3 ± 4.5	5.4 ± 4.6	0.84	5.7 ± 4.5	4.6 ± 4.5	0.02	5.4 ± 4.5	4.8 ± 4.5	6.0 ± 4.6	4.5 ± 4.6	0.08
**NF2**	152 (54.1 %)	121 (51.3 %)	31 (68.9 %)	0.03				109 (58.0 %)	43 (46.2 %)	0.07					
**Supratent**	188 (66.9 %)	152 (64.4 %)	36 (80.0 %)	0.05	109 (72.7 %)	79 (60.3 %)	0.07								
**Ki-67 index**	2.5 ± 2.0	2.2 ± 1.6	3.7 ± 3.2	0.01	2.9 ± 2.4	2.0 ± 1.4	0.004	2.7 ± 2.3	2.0 ± 1.3	0.02	3.2 ± 2.6	2.2 ± 1.4	2.0 ± 1.5	1.8 ± 1.2	8.4×10^{-5}
In GTR	N = 236	N = 209	N = 27		N = 131	N = 105		N = 158	N = 78		N = 90	N = 42	N = 68	N = 36	
**5-yr PFS**	86.9 %				81.5 %	94.1 %	0.02	84.4 %	93.1 %	0.01	77.3 %	93.8 %	94.4 %	92.3 %	6.2×10^{-3}
**Time to recur. (yr)**	4.5 ± 3.7				4.3 ± 3.6	5.3 ± 4.5	0.71	4.6 ± 3.9	4.1 ± 0.1	0.57	4.3 ± 3.7	4.0	5.5 ± 5.0	4.2	0.91
**NF2**	131 (55.5 %)	110 (52.6 %)	21 (77.8 %)	0.01				90 (57.0 %)	41 (52.6 %)	0.57					
**Supratent**	158 (66.9 %)	133 (63.6 %)	25 (92.6 %)	0.001	90 (68.7 %)	68 (64.8 %)	0.57								
**Ki-67 index**	2.5 ± 2.0	2.3 ± 1.6	4.3 ± 3.4	0.001	2.8 ± 2.3	2.1 ± 1.4	0.008	2.7 ± 2.3	2.0 ± 1.2	0.03	3.2 ± 2.6	2.1 ± 1.3	2.2 ± 1.6	1.8 ± 1.2	0.01
**FOXM1 111 cases**	1.1 ± 2.0	1.0 ± 2.0	2.0 ± 2.5	0.17	1.6 ± 2.5	0.5 ± 1.0	0.01	1.5 ± 2.5	0.5 ± 0.9	0.01	2.2 ± 2.9	0.6 ± 0.9	0.6 ± 1.1	0.3 ± 0.9	0.01
In STR	N = 45	N =29	N = 16		N = 20	N = 25		N = 30	N = 15		N = 18	N = 2	N = 12	N = 13	
**5-yr PFS**	65.4 %				55.6 %	75.1 %	0.21	69.3 %	58.2 %	0.61	50.0 %	100 %	100 %	51.3 %	0.05
**Time to recur. (yr)**	3.5 ± 3.7				2.7 ± 2.6	4.6 ± 4.7	0.57	2.9 ± 2.6	4.5 ± 5.0	0.86	2.7 ± 2.6	n/a	5.38	4.5 ± 5.0	0.63
**NF2**	20 (44.4 %)	10 (37.0 %)	10 (55.6 %)	0.2				18 (60.0 %)	2 (13.3 %)	0.004					
**Supratent**	30 (66.7 %)	19 (70.4 %)	11 (61.1 %)	0.53	18 (90.0 %)	12 (48.0 %)	0.004								
**Ki-67 index**	2.3 ± 2.1	1.9 ± 1.7	2.8 ± 2.7	0.42	3.3 ± 2.6	1.5 ± 1.2	0.01	2.4 ± 2.3	2.0 ± 1.7	0.6	3.3 ± 2.6	3.5 ± 3.5	1.2 ± 0.9	1.7 ± 1.4	0.1

Recur.: Recurrence, *NF2*: *NF2* mutation or 22q loss, non-*NF2*: mutation in *AKT1*, *KLF4*, *POLR2A*, *SMO*, and others, Supratent.: Supratentorial lesion, Infratent.: Infratentorial lesion, *NF2*,Sup: "*NF2* + supratentorial lesion", *NF2*, Inf: "*NF2* + infratentorial lesion", n*NF2*, Sup: "non–*NF2* + supratentorial lesion", n*NF2*, Inf: "non–*NF2* + infratentorial lesion", GTR: gross total resection, STR: subtotal resection
